# Multiplexed Imaging Mass Cytometry Analysis in Preclinical Models of Pancreatic Cancer

**DOI:** 10.3390/ijms25031389

**Published:** 2024-01-23

**Authors:** Marco Erreni, Maria Rita Fumagalli, Damiano Zanini, Ermes Candiello, Giorgia Tiberi, Raffaella Parente, Raffaella D’Anna, Elena Magrini, Federica Marchesi, Paola Cappello, Andrea Doni

**Affiliations:** 1 Unit of Multiscale and Nanostructural Imaging, IRCCS Humanitas Research Hospital -, via Manzoni 56, 20089 Rozzano, Milan, Italy; 2 Department of Biomedical Sciences, Humanitas University, Via Rita Levi Montalcini 4, Pieve Emanuele, 20072 Milan, Italy; 3 Department of Molecular Biotechnology and Health Sciences, University of Turin, Piazza Nizza 44b, 10126 Torino, Italy; 4 IRCCS Humanitas Research Hospital -, via Manzoni 56, 20089 Rozzano, Milan, Italy; 5 Department of Medical Biotechnology and Translational Medicine, University of Milan, 20133 Milan, Italy

**Keywords:** imaging mass cytometry, multiplexed imaging, pancreatic cancer, PDAC, preclinical models

## Abstract

Pancreatic ductal adenocarcinoma (PDAC) is one of the most lethal cancers. PDAC is characterized by a complex tumor microenvironment (TME), that plays a pivotal role in disease progression and resistance to therapy. Investigating the spatial distribution and interaction of TME cells with the tumor is the basis for understanding the mechanisms underlying disease progression and represents a current challenge in PDAC research. Imaging mass cytometry (IMC) is the major multiplex imaging technology for the spatial analysis of tumor heterogeneity. However, there is a dearth of reports of multiplexed IMC panels for different preclinical mouse models, including pancreatic cancer. We addressed this gap by utilizing two preclinical models of PDAC: the genetically engineered, bearing *KRAS*–*TP53* mutations in pancreatic cells, and the orthotopic, and developed a 28–marker panel for single–cell IMC analysis to assess the abundance, distribution and phenotypes of cells involved in PDAC progression and their reciprocal functional interactions. Herein, we provide an unprecedented definition of the distribution of TME cells in PDAC and compare the diversity between transplanted and genetic disease models. The results obtained represent an important and customizable tool for unraveling the complexities of PDAC and deciphering the mechanisms behind therapy resistance.

## 1. Introduction

Pancreatic ductal adenocarcinoma (PDAC) represents the seventh most common cause of cancer–related death worldwide. Although relatively rare (2.7% of all cancer cases in 2020, based on World Cancer Research Fund International), PDAC is an extremely lethal disease, with a 5 year survival rate of only 5–10%. PDAC’s poor survival rate is mainly due to the asymptomatic evolution of the disease, which is therefore diagnosed at advanced stages. Upon diagnosis, depending on tumor localization and stage, only 10–20% of PDAC patients are eligible to undergo tumor resection. Surgery is usually combined with chemotherapy (e.g., FOLFIRINOX) and radiotherapy, with a slight improvement in patient survival after treatment [1,2,3].

PDAC carcinogenesis generally results from the progressive accumulation of activating mutations in specific genes, such as *KRAS* and *TP53*, leading to the formation of precursor lesions (pancreatic intraepithelial neoplasia, PanIN), which progressively evolve into invasive PDAC [3]. Besides mutations that modify the morphology of the epithelial tissue, PDAC is characterized by a massive recruitment and activation of cancer–associated fibroblasts (CAFs) and deposition of extracellular matrix (ECM) components, leading to a desmoplastic reaction that profoundly modifies the surrounding connective tissue [4]. The resulting tumor stroma, which can develop up to 90% of the entire tumor mass, consists of a dense ECM, infiltrated by immune cells, CAFs and endothelial cells (ECs), that actively interacts with PDAC cells, influences tumor prognosis, and contributes to the resistance to anti–tumor treatments [5,6,7]. Given the importance of the tumor microenvironment (TME) in PDAC progression and resistance to therapy, several strategies aimed at deconstructing the tumor stroma or targeting immunosuppressive pathways have been tested. The ineffective results obtained underline the need to investigate more deeply the composition of the TME and its role in tumor progression [8,9].

The use of preclinical mouse models has been widely applied to investigate the complexity of the TME and to evaluate the efficacy of new anti–tumor therapeutic approaches. In this context, several PDAC mouse models, recapitulating features of the human disease, have been developed, including transplanted orthotopic tumors and genetically engineered models [1,10]. The Panc02 cell line is one of the most commonly used to generate PDAC transplanted mouse models: orthotopic injection of Panc02 cells results in the formation of a well–differentiated tumor, characterized by a high resistance to a wide range of chemotherapeutic drugs and a less pronounced desmoplastic reaction, which mainly surrounds the tumor mass [11,12]. In addition, one of the most sophisticated genetic models of PDAC is represented by *LSL*–*Kras^G12D/+^*, *LSL*–*Trp53^R172H/+^*, and *Pdx1*–*Cre* (KPC) mice [13]. KPC mice express mutant isoforms of *KRAS* and *TP53* genes specifically in pancreatic cells, leading to the formation of PanIN and, subsequently, PDAC in immune–competent mice. Histologically, KPC mice show pronounced desmoplasia, low immune infiltration, poor angiogenesis and high metastatic burden, closely resembling human disease [14].

For a more complete dissection of the cellular and molecular actors of the TME, fluorescence– and non–fluorescence–based multiplex imaging techniques, that allow simultaneous visualization of multiple cell markers on the same tissue section, have been recently developed [15]. These include single–cell imaging mass cytometry (IMC), a technology that combines conventional histology with mass cytometry to detect up to 40 different metal–tagged antibodies, overcoming limitations frequently observed with fluorescence–based tissue imaging, such as tissue autofluorescence and spectral overlap [16,17]. Within the tissue section, selected regions of interest (ROIs) are ablated with a UV laser with a spatial resolution of 1 μm^2^ and metal tags are detected by a time–of–flight (TOF) mass spectrometer, with a resolution of 1 Da. Single–cell segmentation is then applied to acquired images, in order to identify single cells and analyze their localization within the tissue, their connection with neighboring cells and their expression of each marker [18,19].

In the last decade, IMC has been widely applied to analyze TME heterogeneity in a variety of human tissue samples, but only a few studies have been performed on mouse models of carcinogenesis. In this manuscript, we established and validated a novel IMC panel comprising 28 metal–tagged antibodies. These antibodies were specifically designed for the examination of the TME and the analysis of immune cell composition within frozen tissue sections of murine models of pancreatic cancer. This specialized panel was deployed to assess the TME composition in PDAC using both the Panc02 orthotopic and KPC mouse models, with the aim of comparing these mouse models and assessing their apparent similarities and differences with human disease.

## 2. Results

### 2.1. Study of the TME in an Orthotopic Model of PDAC Using IMC

We first selected 28 markers to completely reconstruct the cellular landscape of PDAC. The antibody list is shown in Table 1 and includes markers to identify epithelial and tumor cells (PanCK, E–cadherin, CK19, and ZO–1), blood (CD31 and PV–1) and lymphatic (LYVE–1) ECs, stromal cells and ECM components (Vimentin, fibrinogen, αSMA, PDGFRα, PDGFRβ, uPAR, Desmin, Collagen I, Collagen IV and CD44). Regarding the immune compartment, the IMC panel comprises a pan–leukocyte marker (CD45) and antibodies to specifically identify neutrophils (Ly6G), monocytes (CD11b), macrophages (F4/80), T cells (CD3, CD4, and CD8), dendritic cells (CD103) and B cells (B220). Additional markers were included to characterize certain functions of the infiltrating immune cells, such as the M1/M2 macrophage ratio (CD206) and activation/antigen presentation (MHC–II). An iridium–based DNA intercalator (Ir–191/193, Standard Biotools) was used to identify nuclei. 

**Figure 1:**
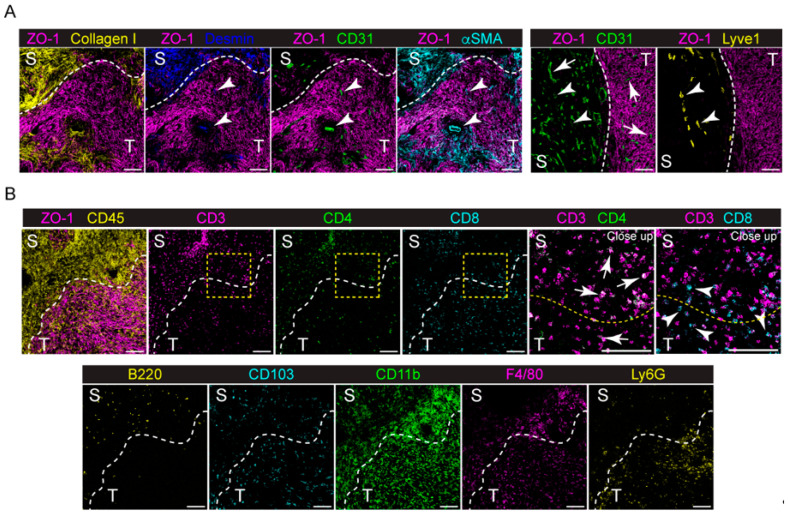
Expression of different markers at the tumor–stroma interface of the orthotopic PDAC model detected by IMC. (**A**) Left panel, representative images showing the extracted signal contributions of Collagen I (yellow), Desmin (blue), CD31 (green), αSMA (cyan) and ZO–1 (magenta). White arrowheads indicate blood vessels (CD31^+^, green) colocalizing with Desmin^+^ and αSMA^+^ cells. Right panel, representative images showing the signal contributions of CD31^+^ (green), LYVE1^+^ (yellow) and ZO–1^+^ cells. White arrowheads, lymphatic vessels; white arrows, blood vessels. (**B**) Upper, left, representative images showing the signal contributions of ZO–1^+^ (magenta), CD45^+^ (yellow), CD3^+^ (magenta), CD4^+^ (green) and CD8^+^ (cyan) cells. Upper, right, close up images of the yellow dashed inset showing the different localizations of CD3^+^ (magenta) and CD4^+^ (green) cells (white arrows) compared to CD3^+^ (magenta) and CD8^+^ (cyan) cells (white arrowheads). Lower, representative images showing the localization of B220^+^ (yellow), CD103^+^ (cyan), CD11b^+^ (green), F4/80^+^ (magenta) and Ly6G^+^ (yellow) cells. The dashed white line delineates stromal–rich (S) and tumor–cell–rich (T) regions. Bar, 100 μm.

We first used the panel to investigate the TME of the Panc02–model orthotopic mouse model of PDAC. For this purpose, syngeneic Panc02 cells (1 × 10^6^) were injected into the head of the pancreas of C57B/6 mice (*n* = 4), as described in Section 4. Tumors were collected on day 21 after tumor cell injection and stained with the IMC panel. As shown in Figure 1 and Supplementary Figure S1, the Panc02 tumor was grown as a dense mass of ZO–1^+^ tumor cells, surrounded by a dense stromal tissue, mainly composed of Collagen I, Desmin and αSMA^+^ cells (Figure 1A, left panel). Analyzing the tumor–stroma interface, we observed that the majority of CD31^+^ blood ECs are located in the surrounding stromal tissue capsule (Figure 1A, left and right panels), while only few of them can be identified in the tumor tissue, colocalizing with Desmin^+^ and αSMA^+^ cells (Figure 1A, left panel), By contrast, LYVE–1^+^ lymphatic vessels (LVs) are located only in the surrounding stroma (Figure 1A, right panel). Immune cells highly infiltrate the tumor (Figure 1B), with subpopulations showing differences in localization. In particular, CD3^+^ CD4^+^ T cells seem to be mainly located in the stromal tissue (Figure 1B, inset), whereas CD3^+^ CD8^+^ T cells are more prone to infiltrate the tumor (Figure 1B, inset). Similarly, B220^+^ B cells infiltrate the surrounding stroma, while CD11b^+^ F4/80^+^ monocytes/macrophages penetrate more into the tumor mass. Ly6G^+^ neutrophils are mainly located along the tumor–stroma interface, while CD103^+^ dendritic cells (DCs) are equally scattered between the tumor and stromal tissue (Figure 1B).

**Figure 2:**
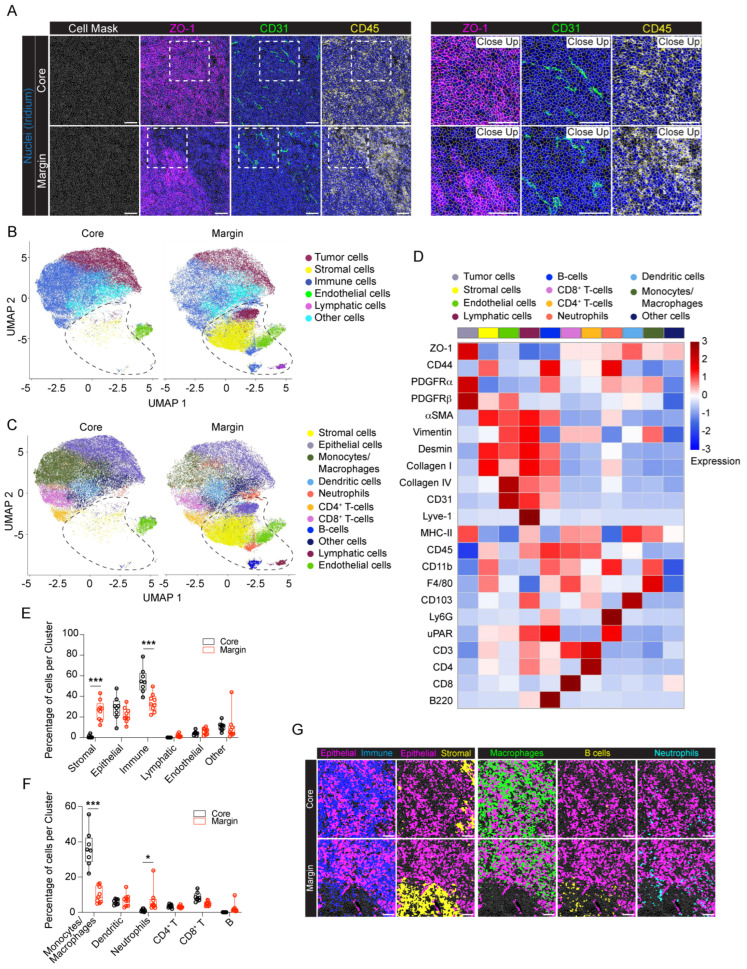
IMC analysis of the orthotopic PDAC model. (**A**) Representative images (**left**) and close up images (**right**) of cell segmentation in the tumor core and margin. The cell contour (gray) is overlaid on the IMC acquisition of nuclei (Ir, blue) and ZO−1^+^ tumor cells (magenta), CD31^+^ endothelial cells (green) and CD45^+^ immune cells (yellow). Bar 100 µm. (**B**) UMAP representation, over all the acquired images, of tumor core and tumor margin cells annotated into 6 phenotypic clusters, as in the legend. The dashed black lines indicate cells mainly associated with the stromal tissue surrounding the tumor mass. (**C**) The same UMAP representations as in panel (**B**) showing the detailed annotation of immune cell subpopulations: monocytes/macrophages, dendritic cells, neutrophils, CD4^+^ T cells, CD8^+^ T cells and B cells. Colors as in the legend. The dashed black lines indicate cells mainly associated with the stromal tissue surrounding the tumor mass. (**D**) Heatmap of the mean signal intensity of single markers among the different clusters over all the acquired images. (**E**) Abundance of cells per phenotypic cluster for the tumor core and tumor margin. Dots represent single ROIs. Colors as in the legend. (**F**) Abundance of cells in the immune clusters for the tumor core and tumor margin. Dots represent single ROIs. Colors as in the legend. (**G**) Representative images showing the tissue localization of segmented cells (gray contours) belonging to epithelial (magenta), immune (blue), stromal (yellow), macrophage (green), B cell (yellow) and neutrophil (cyan) clusters in the tumor core and tumor margin. Bar, 100 μm. *n*= 3 to 6 ROIs for each mouse (*n* = 4). Total acquired ROIs: core, *n* = 8; margin*, n* = 9. (**E**,**F**) Two-way ANOVA plus Šídák’s multiple comparisons test. * *p* < 0.05, *** *p* < 0.001.

**Figure 3:**
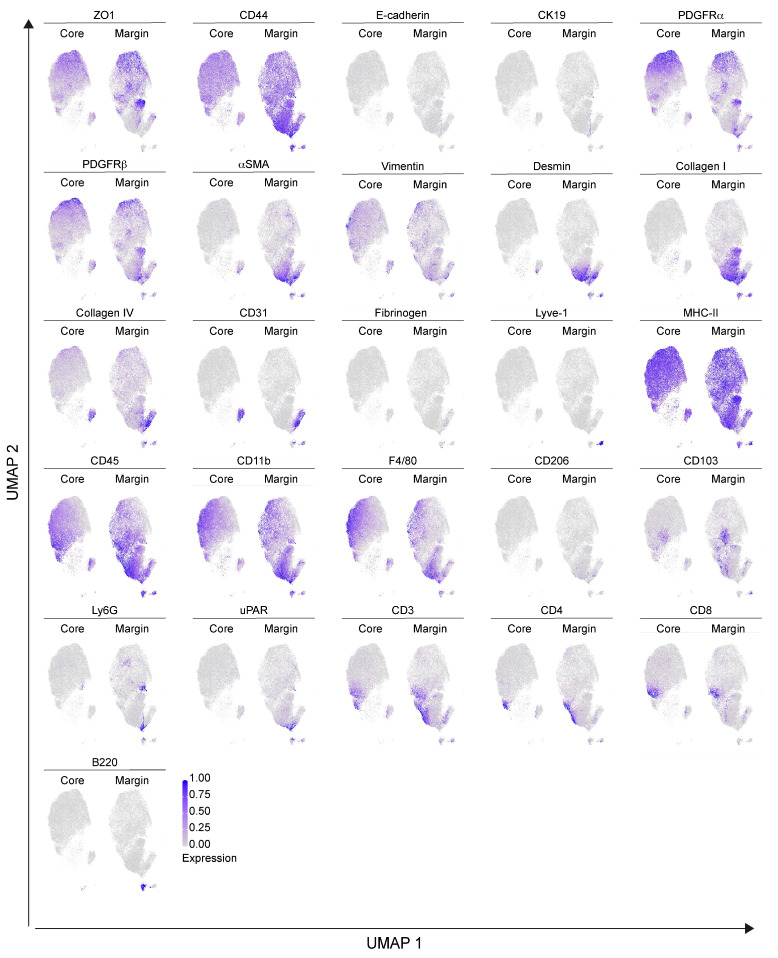
Single marker expression in the orthotopic PDAC model. Single markers’ normalized expression in all the segmented cells from the orthotopic model represented on UMAP. *n* = 3 to 6 ROIs for each mouse (*n* = 4). Total acquired ROIs: core: *n* = 8; margin: *n* = 9.

To dissect the cellular composition and distribution in the TME of the Panc02 orthotopic model of PDAC, we evaluated the inner part of the tumor mass (core) with the tumor–stromal interface (margin) for comparative analyses. We randomly selected eight and nine regions of interest (ROIs) for the tumor core and tumor margin, respectively. Single–cell segmentation was performed as described in detail in Section 4. Figure 2A shows a representative ROI of the generated cell masks in the tumor core and margin, overlaid on the IMC signal of ZO–1^+^ tumor cells, CD31^+^ ECs and CD45^+^ immune infiltrating cells. Among the 28 markers included in the IMC panel, only PanCK and PV1 were excluded from the analysis, due to unspecific and low signal detection, respectively. A total number of *n* = 78,116 cells were identified, *n* = 36,677 (47%) belonging to the tumor core and *n* = 41,439 (53%) belonging to the tumor margin. We then used Ly6G, Vimentin, CD45, αSMA, F4/80, PDGFRα, PDGFRβ, CD3, CD4, CD8, Lyve–1, Desmin, CD31, Collagen IV, Collagen I, CD11b, ZO–1, CD103, and B220 markers for UMAP reduction and cell clustering. Phenograph analysis identified 19 different clusters that, based on the expression of specific markers, were annotated as 6 clusters representing endothelial (5%), tumor (25%), immune (44%), lymphatic (1%), stromal (14%) and other cells (10%) (Figure 2B). The expression of the single markers in the phenograph analysis shows the good quality of the clustering procedure (Figure 3). The immune cell clusters contained six distinct clusters, corresponding to monocytes/macrophages, DCs, neutrophils, CD4^+^ T cells, CD8^+^ T cells and B cells (Figure 2C,D). The heatmap in Figure 2D shows the expression of the 26 markers per cluster. Tumor cells express high levels of ZO–1, as well as the mesenchymal markers PDGFRα and PDGFRβ, underlying the invasive phenotype of the Panc02 cells. Stromal compartment results enriched in CD44, αSMA, Desmin and Collagen I. CD31^+^ Collagen IV^+^ ECs and LYVE–1^+^ lymphatic ECs are associated with stromal makers, including αSMA, Desmin and Vimentin, thus suggesting that blood vessels and LVs are closely surrounded by stromal tissue, as already shown in Figure 1A. Finally, specific markers clearly correlate to their corresponding immune cell cluster, such as CD103 for DCs, B220 for B–lymphocytes, CD3–CD4 and CD3–CD8 for T cells, F4/80 for macrophages, and Ly6G for neutrophils (Figure 2D). Among the 26 markers, fibrinogen, CD206, and E–cadherin were excluded from the heatmap because of their low expression. In spite of that, the phenograph analysis shows that CD206^+^ and fibrinogen^+^ clusters correspond to F4/80^+^ ECM remodeling macrophages and CD31^+^ ECs, respectively, indicating M2–polarized macrophages and fibrinogen–associated blood vessels (Figure 3).

As revealed by the phenograph analysis, we selectively identified a group of cells associated with the tumor margin (Figure 2B and Supplementary Figure S2A) and predominantly belonging to the stromal cell cluster (core 0.93% vs. margin 26.30%; *p* < 0.001). In line with this observation, stromal markers, such as αSMA, Desmin, Collagen I, and CD44, were more expressed at the tumor margin compared to the tumor core (Supplementary Figure S2B). Similarly, the LV cluster (core 0.02% vs. margin 1.87%; *p* = 1) (Figure 2B,E) was predominantly associated with the tumor margin, rather than the tumor core. In general, immune cells were instead associated with the tumor core more than the margin (core 55.40% vs. margin 33.83% *p* < 0.001), even if specific differences in the location of the different cell subtypes were observed (Figure 2B,C,E,F). Specifically, monocyte/macrophages (core 36.18% vs. margin 9.85%; *p* < 0.001), as well as CD8^+^ T cells (core 8.69% vs. margin 4.95%; *p* = 0.32), were mostly located in the tumor core (Figure 2F,G). In addition, monocytes/macrophages and CD8^+^ T cells at the tumor margin expressed higher levels of MHC–II and CD44, respectively, reflecting a different state of inflammatory activation of these cells (Supplementary Figure S2C,D). By contrast, neutrophils and B cells were mainly located at the tumor margin (neutrophils: core 1.08% vs. margin 6.69%, *p* = 0.03; B cells: core 0.07% vs. margin 2.25%; *p* = 0.85) (Figure 2F,G), whereas no difference was found in DC and CD4^+^ T cell distribution between the core and margin of the tumor (DCs: core 5.89% vs. margin 6.93%, *p* = 1; CD4^+^ T cells: core 3.49% vs. margin 3.15%, *p* = 1). Finally, although ECs are equally distributed between the tumor core and margin, indicating a similar vascularization, the expression of αSMA and Collagen IV in ECs is higher at the tumor margin, therefore suggesting differences in blood vessel stabilization and functionality between the tumor margin and core (Supplementary Figure S2E).

**Figure 4:**
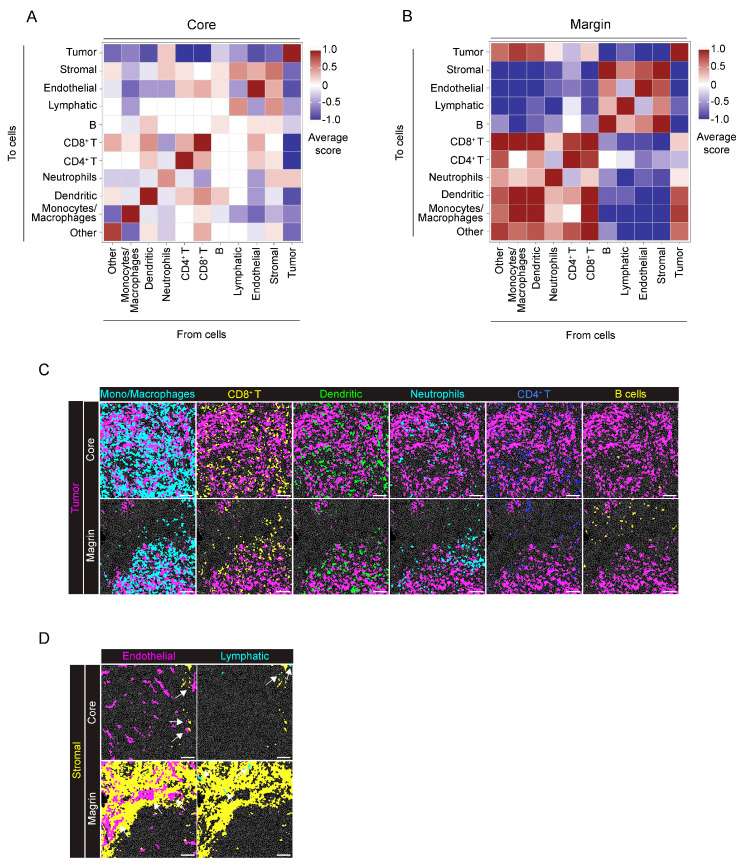
Neighborhood analysis of the orthotopic PDAC model. (**A**) The heatmap shows the average proximity score for each pair of cell phenotypic clusters, averaged over all the acquired ROIs (8 ROIs). Positive (red) or negative (blue) values indicate that a specific pair of phenotypes is neighboring significantly more often or significantly less often, respectively, than expected from randomized placement, as described in Section 4. A 30 μm radius is considered for cell−to−cell proximity. (**B**) Tumor margin, as in panel (**A**), averaged over all the acquired ROIs (9 ROIs). A 30 μm radius is considered for cell−to−cell proximity. (**C**) Representative images showing the distribution of cells belonging to the monocyte/macrophage (cyan), CD8^+^ T cell (yellow), dendritic cell (green), neutrophil (cyan), CD4^+^ T cell (blue) and B cell (yellow) clusters with the tumor cell (magenta) cluster over the cell mask, in the tumor core and margin. (**D**) Representative images showing the arrangement of cells belonging to the endothelial (magenta) and lymphatic (cyan) clusters in relation to the stromal cluster (yellow) in the tumor core and tumor margin, white arrows: Endothelial, green arrowss: Lymphaic. Bar, 100 μm. *n* = 3 to 6 ROIs for each mouse (*n* = 4). Total acquired ROIs: core: *n* = 8; margin: *n* = 9.

In neighborhood analysis (proximity range of 30 µm radius from cell borders), as expected from the growth pattern of the tumor cells in the model, Panc02 cells were closely associated with themselves and not grouped with any other cells, except for a slight association with neutrophils (Figure 4A). By contrast, at the tumor margin, two different clusters of associated cells were specifically identified (Figure 4B) and correspond to mono–macrophages, neutrophils, dendritic cells and CD8^+^ T cells, indicating a proximity and functional interaction of these immune cells (Figure 4B,C). Conversely, CD4^+^ T cells and B cells were located far from the tumor mass (Figure 4B,C). The second cluster is related to stromal cells, ECs and lymphatic cells, indicating the localization of blood and LVs within the tumor–surrounding ECM (Figure 4B,D). Notably, these results are in line with the stromal markers associated with CD31^+^ and LYVE−1^+^ cells, as previously shown in the heatmap (Figure 2D).

Therefore, the development and application of an extensive antibody panel combined with IMC stand out as a crucial methodology for the assessment of localization and distribution of different cell subtypes comprising the TME in PDAC. IMC analysis enables exhaustive delineation of the distinct cell distribution patterns interacting with PDAC.

### 2.2. Study of the TME Genetic Model of PDAC Using IMC

**Figure 5:**
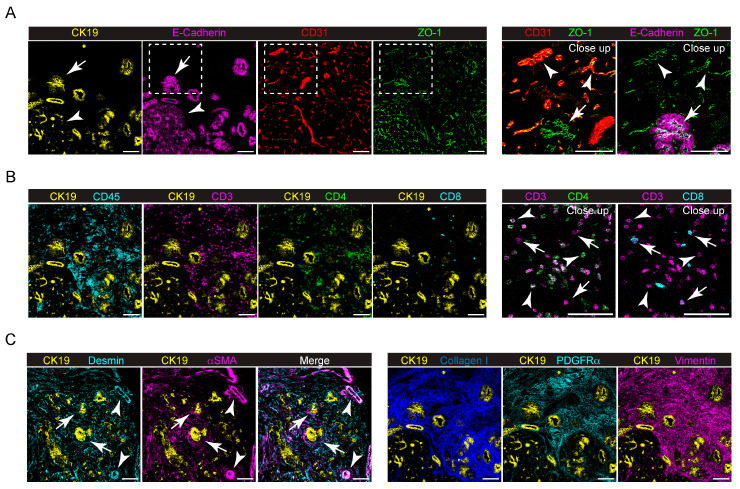
Expression of different markers in the KPC genetic model detected by IMC. (**A**) Left, representative images showing the extracted signal contributions of CK19^+^ (yellow), E−cadherin^+^ (magenta), CD31^+^ (red) and ZO−1^+^ (green) cell signals. Right, close−up of the white dashed boxes showing the colocalization of ZO−1^+^ cells with CD31^+^ (white arrowheads) and E−cadherin (white arrows). (**B**) Left, representative images showing CK19^+^ (yellow) cell distribution with CD45^+^ (cyan), CD3^+^ (magenta), CD4^+^ (green) and CD8^+^ (cyan) cell signals in the KPC model. Right, the close−up shows the identification of CD3^+^ CD4^+^ (white arrowheads) and CD3^+^ CD8^+^ (white arrows) cells. (**C**) Left, representative images showing the colocalization of CK19^+^ (yellow), Desmin^+^ (cyan) and αSMA^+^ (magenta) cells in KPC. White arrowheads indicate Desmin^+^ and αSMA^+^ cells surrounding CK19^+^ tumor cells. White arrows indicate Desmin^+^ and αSMA^+^ cells surrounding tumor vessels. Right, expression of Collagen I (blue), PDGFRα^+^ (cyan) and Vimentin^+^ (magenta) cells in KPC, surrounding CK19^+^ tumor cells (yellow). Bar, 100 μm.

We then applied the IMC panel and computing analysis to study the PDAC microenvironment in a genetic mouse model of the disease. To this end, we used the *LSL*–*Kras^G12D/+^*, *LSL*−*Trp53^R172H/+^*, and *Pdx1*−*Cre* (KPC) mouse models, which express mutant isoform of *KRAS* and *TP53* specifically in pancreatic cells, leading to PDAC onset in immune–competent mice. Frozen sections from two KPC mice (KPC1 and KPC2, respectively) were stained with the IMC panel. All the 28 markers included in the IMC panel provided specific signals and were therefore included in the analysis (Figure 5 and Supplementary Figure S3). Tumor cells resulted positive for the epithelial markers CK19 and E−cadherin (Figure 5A) and Pan–cytokeratin (Supplementary Figure S3). Notably, CK19 and E−cadherin colocalized in tumor cells (Figure 5A, left panel), while in the adjacent healthy tissue, CK19 and E−cadherin expression was restricted to ductal cells and exocrine pancreatic cells, respectively (Figure 5A left panel). CD31 identified blood vessels infiltrating the tumor tissue and colocalized with the tight−junction molecule ZO−1 (Figure 5A). A different expression and distribution of ZO−1 (Figure 5A) would suggest, as previously reported [20], the heterogeneity of tumor blood vessels, from a functional to a non−functional leaking vasculature. ZO−1 also colocalized with CK19^+^ E−cadherin^+^ tumor cells (Figure 5A). In contrast with the previous orthotopic model, CD45^+^ immune cells abundantly infiltrated the tumor tissue (Figure 5B), with CD3^+^ T cells being the most important immune cell subtype. In particular, CD3^+^ CD4^+^ T cell infiltration was more prominent compared to that of CD3^+^ CD8^+^ cytotoxic T cells (Figure 5B). Tumor cells were immersed in a dense stromal ECM, as suggested by the relevant expression of Desmin, αSMA, Collagen I, PDGFRα and Vimentin (Figure 5C). Notably, Desmin^+^ and αSMA^+^ cells were located both around vessel structures and in the ECM, likely indicating the presence of specialized cancer−associated fibroblasts (CAFs) surrounding CK19^+^ neoplastic cells (Figure 5C). PDGFRα^+^ and Vimentin^+^ stromal cells were associated with an organized collagen−rich desmoplastic ECM (Figure 5C).

**Figure 6:**
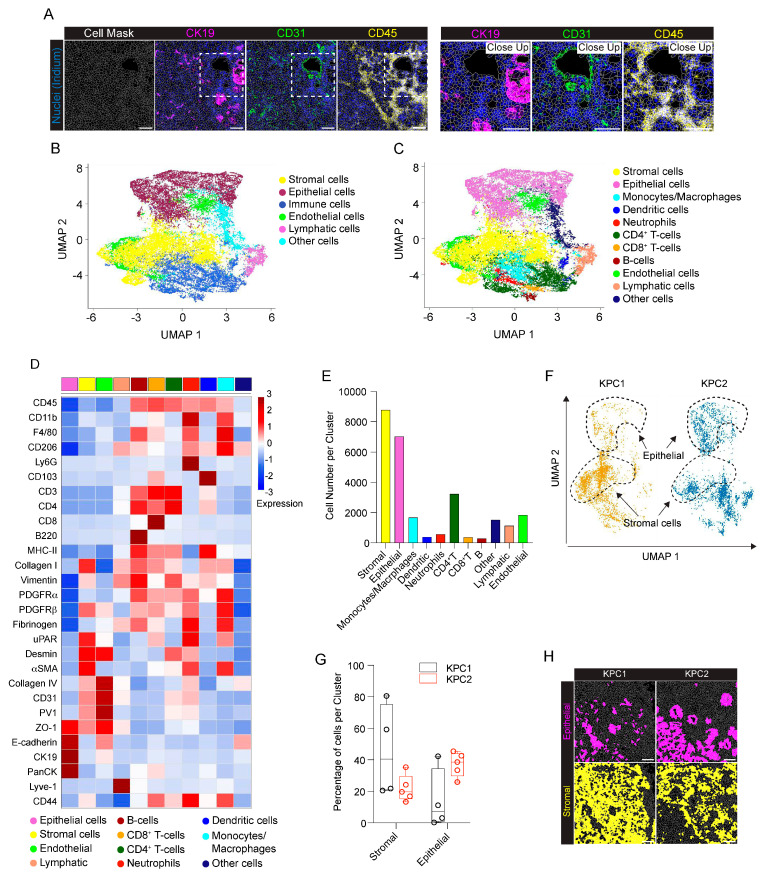
IMC analysis of the genetic KPC model. (**A**) Representative images of cell segmentation in the KPC model. The cell mask (gray lines) is overlaid to the IMC acquisition of nuclei (Ir, blue) and CK19^+^ tumor cells (magenta), CD31^+^ endothelial cells (green) and CD45^+^ immune cells (yellow). Right, close up images of the white dashed region in A. Bar, 100 μm. (**B**) UMAP representation of the KPC model segmented cells annotated as 6 phenotypic clusters. Colors as in the legend. (**C**) Detailed annotation of immune cell subpopulations, namely monocytes/macrophages, dendritic cells, neutrophils, CD4^+^ T cells, CD8^+^ T cells and B cells (colors as in the legend) represented on the same UMAP reduction as in panel (**B**). (**D**) Heatmap of the average expression of the single markers among the different clusters, over all the acquired images. (**E**) The bar plot shows the number of annotated cells for every identified cluster. Colors as in the legend. (**F**) UMAP representation of segmented cells of two different ROIs from KPC1 and KPC2 mice. Black−dashed lines delimit the regions corresponding to the localization of epithelial (top) and stromal (bottom) phenotypic clusters. (**G**) The box plot shows the difference in the number of cells in the stromal and epithelial clusters between KPC1 and KPC2 mice. Dots represent single ROIs. Colors as in the legend. Stromal: *p* = 0.12; epithelial: *p* = 0.13, two−way ANOVA plus Šídák’s multiple comparisons test. (**H**) Representative images of the different distribution of cells belonging to epithelial (magenta) and stromal (yellow) clusters in KPC1 and KPC2 mice. Scalebar: 100 μm. *n* = 4 to 5 ROIs for each mouse (*n* = 2). Total acquired ROIs: KPC1, *n* = 4; KPC2, *n* = 5.

**Figure 7:**
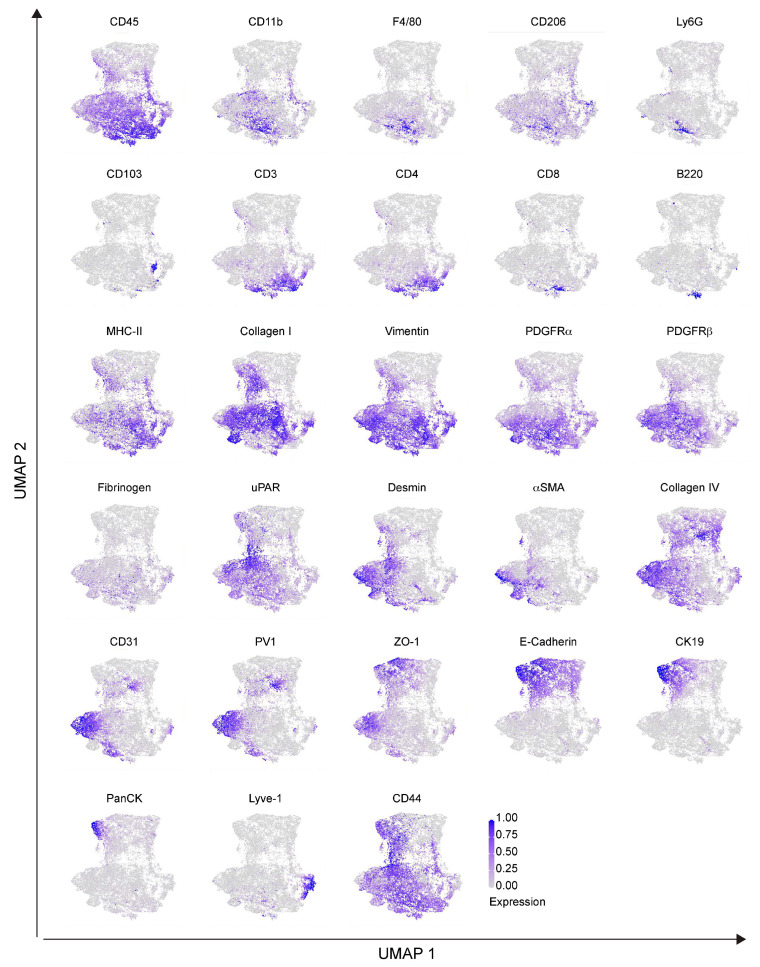
Single marker expression in the genetic KPC model. UMAP representation of single markers’ normalized expression in all the segmented cells from the KPC model. *n* = 4 to 5 ROIs for each mouse (*n* = 2). Total acquired ROIs: KPC1, *n* = 4; KPC2, *n* = 5.

For the IMC phenotypical analysis of the KPC model, we selected four ROIs from KPC1 and five ROIs from KPC2. A representative cell mask is shown in Figure 6A and the corresponding inset, overlaid with the IMC signal detected for CK19^+^ tumor cells, CD31^+^ ECs and CD45^+^ immune cells. A total number of 26,861 cells were identified (KPC1: 12,249; KPC2: 14,612). We then used Ly6G, Vimentin, CD45, PanCK, αSMA, CK19, F4/80, PDGFRα, PDGFRβ, E−Cadherin, CD3, CD4, CD8, Lyve−1, Desmin, CD31, Collagen IV, Collagen I, ZO−1, CD103, and B220 as markers for UMAP reduction and cell clustering. Phenograph analysis identified 20 clusters that, based on the expression of specific markers, were annotated as 6 clusters corresponding to epithelial (26%), stromal (33%), immune (24%), ECs (7%), lymphatic (4%) and other cells (6%) (Figure 6B). The immune cell cluster contained 6 distinct clusters, corresponding to monocyte/macrophages (6%), DCs (1%), neutrophils (2%), CD3^+^ CD4^+^ T lymphocytes (12%), CD3^+^ CD8^+^ T lymphocytes (1%) and B lymphocytes (1%) (Figure 6C–E). As revealed by the heatmap (Figure 6D), a clear clustering of epithelial−derived tumor cells that express ZO–1, E–cadherin, CK19, and PanCK was identified (Figure 6D). The expression of each single marker in the phenograph clustering indicates the presence of two different subpopulations of epithelial cells, expressing either E–cadherin alone or the combination of E–cadherin, CK19, and PanCK, which provides distinct evidence of differences between parenchymal epithelial cells and tumor cells (Figure 7). Therefore, this result is in line with the IMC staining shown in Figure 5A, where tumor cells expressing CK19, E−cadherin and PanCK were close to healthy exocrine epithelial cells expressing E−cadherin alone. A cluster of ECs was identified in the tumor by the expression of CD31, ZO−1, PV1 and Collagen IV (Figure 6D). In addition, UMAP representation enables the identification of two different groups of CD31^+^ ECs, associated with either the stromal or the epithelial clusters (Figure 7). Moreover, while Collagen IV and PV1 are equally expressed in both CD31^+^ cell clusters, ZO−1 expression is mainly associated with that related to the stromal cell cluster. Immune cell markers of myeloid and lymphoid subtypes identified clusters of CD103^+^ DCs, B220^+^ B−lymphocytes, CD3^+^CD4^+^ and CD3^+^CD8^+^ T cells, F4/80^+^ macrophages and Ly6G^+^ neutrophils (Figure 6D). In the monocyte/macrophage cluster, the expression of both MHC−II and CD206 markers was found, suggesting the presence of tumor−associated macrophages with different polarization states. The expression of ECM components, including Collagen I, fibrinogen, Desmin and Vimentin was weakly spread among both the different immune cell clusters and the PDGFRβ^+^, αSMA^+^ stromal cells cluster, thus suggesting that TME−constituting cells were immersed in the tumor ECM, some of these with (uPA/uPAR) fibrinolytic activity of remodeling of the coagulative stroma (Figure 6D).

By analyzing KPC1 and KCP2 tumors separately, we observed a different positioning of cells in the UMAP, suggesting a reduced number of KPC1 cells in the epithelial cluster region, together with cell enrichment in the stromal cluster (Figure 6F and Supplementary Figure S4A). This observation was confirmed by counting the number of cells per cluster (KPC1: epithelial 14%; stromal 45%; KPC2: epithelial 37%; stromal 23%) (Figure 6G). An example of the spatial distribution of cells belonging to the epithelial or stromal cluster is shown in Figure 6H. Accordingly, Vimentin and Collagen I, as well as epithelial markers, such as CK19, E−cadherin and ZO−1, were differently expressed in KPC 1 and KPC2 (Supplementary Figure S4B,C). In addition, the amount of monocyte/macrophage infiltrating the tumor tissue was higher in KPC1 compared to KPC2 (KPC1 12% vs. KPC2 1%, *p* < 0.01), while no difference was observed for the other immune cell subpopulations (Supplementary Figure S4D).

**Figure 8:**
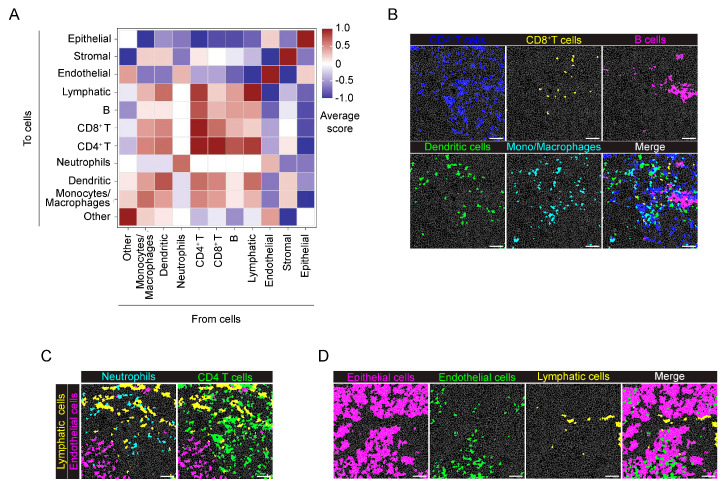
Neighborhood analysis of the genetic KPC model. (**A**) The heatmap shows the proximity score for each pair of cell phenotypic clusters, averaged over all the acquired ROIs (*n* = 9). Positive (red) or negative (blue) values indicate that a specific pair of phenotypic classes is neighboring significantly more or significantly less often, respectively, than expected from randomized placement. A 30 μm radius was considered for cell−to−cell proximity. (**B**) Representative images showing the localization of cells belonging to the CD4^+^ T cell (blue), CD8^+^ T cell (yellow), B cell (magenta), dendritic (green) cell and monocyte/macrophage (cyan) clusters over the cell mask (gray lines). The merged image highlights the close proximity of these immune cell clusters. (**C**) Representative images showing the localization of cells belonging to lymphatic (yellow), endothelial (magenta), neutrophil (cyan) and CD4^+^ T cell (green) clusters over the cell mask. (**D**) Representative images showing the localization of cells belonging to epithelial (magenta), endothelial (green) and lymphatic (yellow) clusters over the cell mask. Gray lines depict single cell contours. Bar, 100 μm. *n* = 4 to 5 ROIs for each mouse (*n* = 2). Total acquired ROIs: KPC1, *n* = 4; KPC2, *n* = 5.

Finally, neighborhood analysis showed that immune cells, such as B cells, CD4^+^ and CD8^+^ T cells, DCs and monocyte/macrophage were spatially associated with the stromal tissue, but separated from the epithelial cells (Figure 8A,B). Immune cells, in particular CD4^+^ T cells and DCs, were in the proximity of LVs but not blood ECs (Figure 8C). Indeed, ECs and lymphatic cells showed a different localization in tissue: blood ECs localized close to epithelial cells, whereas LVs were apparently isolated (Figure 8D). 

In summary, these findings suggest that the IMC panel effectively delineates the differences among the pancreatic cancer models used. Moreover, the IMC analysis elucidates the heterogeneity of these models, in terms of cell abundance, distribution and the reciprocal interplay between cells in the TME and cancer cells.

## 3. Discussion

In the present manuscript, we validated a 28−antibody panel for the IMC analysis of frozen sections of mouse pancreatic cancer. Multiplexed images acquired by IMC allowed us to study the tissue architecture, describing different features of tumor cells, the composition of the desmoplastic stroma and the tumor infiltration by different immune cell populations. Neighborhood analysis implemented phenotypic data by providing unprecedented in situ information on pancreatic cancer, revealing cell distribution and spatial relationships within tumor tissue. The comparative analyses, conducted in both an orthotopic and a genetically−based model, provide knowledge about the diversity of cell abundances and their distribution in the tumor microenvironment.

Compared to other multiplex immunofluorescence imaging techniques, IMC overcomes intrinsic limitations related to the use of fluorescence, including spectral overlap, iterative immunostaining and tissue autofluorescence [15]. For these reasons, IMC has been widely used in many studies aimed at dissecting the composition of different types of human cancer [21,22]. By contrast, only a few studies report the use of IMC in preclinical models of diseases [23,24,25,26,27]. 

The IMC panel was validated in two different PDAC mouse models, a Panc02–cell orthotopic transplanted model and the KPC genetic model. Mouse models of PDAC are widely used to investigate mechanisms driving tumor onset and progression, as well as the efficacy of therapeutic approaches [10,28]. One of the main features of PDAC is represented by the complexity of the TME, which is characterized by the presence of a dense desmoplastic stroma that surrounds tumor cells, preventing immune cell infiltration, activation and the delivery of therapeutic agents [29]. Several studies have described different aspects of the PDAC TME, including the phenotype of cancer−associated fibroblasts and infiltrating immune cells [5,6]. However, only a few of them applied multiplexed imaging technology to unveil the complexity of the pancreatic TME [30,31]. 

By IMC analysis, we showed that a Panc02−derived tumor grows as a dense mass of ZO−1 positive tumor cells, largely infiltrated by immune cells. Histologically, the Panc02−derived tumors do not develop a prominent desmoplastic reaction as observed in human disease [12]. Indeed, by comparing the tumor core and tumor margin, we showed that cells expressing stromal markers, such as Desmin, Collagen I and αSMA, are mainly located in the periphery of the tumor, surrounding the neoplastic mass. On the contrary, we showed that in the KPC model, the desmoplastic stroma is much more abundant (33% of stromal cells versus 14% in the orthotopic model) and evident even inside the mass and characterized by the presence of cells expressing markers typical of CAFs, such as Vimentin, Desmin, PDGFRβ and αSMA [32,33]. Notably, αSMA has been reported to identify a specific CAF population, named myCAFs, which represents up to 50% of CAFs and is mainly responsible for desmoplasia [6,34]. 

Through the IMC analysis of KPC mice used in this study, we found that KPC1 and KPC2 mice show differences in the stromal cell compartment, with KPC2 showing a higher stromal component compared to KPC1, thus indicating that the markers included in the IMC panel effectively discriminate between tumors with different grades of desmoplasia. Indeed, the KPC1 tumor was isolated from a mouse sacrificed at 5 months of age while the KPC2 was from one sacrificed at 7 months of age. The longer survival compared to the median [35] could be explained by the massive infiltration of immune cells into the tumor, but also be the responsible for the more abundant desmoplastic reaction compared the KPC1 tumor.

The excessive deposition of ECM is often associated with vasculature collapse and high interstitial pressure, limiting drug delivery [36,37]. IMC analysis showed that, in the orthotopic model, CD31^+^ blood vessels are mainly located at the periphery of the tumor. In addition, we found that Collagen IV, a basement membrane protein essential for blood vessel stability, is more related to blood vessels located in the tumor surrounding the stroma rather than in the tumor mass. In the genetic model, blood vessels are generally more associated with tumor cells. Markers indicating vessel stability, such as Collagen IV and PV1, are homogeneously expressed, while two different CD31^+^ EC populations, expressing different levels of ZO–1, can be identified, underlying the continuous angiogenic process and the heterogeneity of the tumor vasculature.

Both the orthotopic and genetic model are infiltrated by immune cells. Also in this case, IMC analysis revealed differences in the spatial distribution and abundance of immune cell subpopulations in the two pre−clinical models. In the orthotopic model, the tumor core was infiltrated by macrophages and CD8^+^ T cells, without any specific spatial association with other cell types. By contrast, at the tumor–stromal interface, neighborhood analysis indicated a spatial clustering of immune cells, including CD8^+^ and CD4^+^ T cells, neutrophils, DCs and macrophages. A similar spatial association between CD8^+^ and CD4^+^ T cells, DCs and macrophages has also been found in the KPC genetic model. By contrast, KPC tumors displayed only 24% of immune cells, compared to the 44% observed in the transplantable model. Tumor infiltration by T cells has been investigated in several studies, generally associated with improved overall survival [38]. In addition, DCs and macrophages are in proximity to tumor cells. Tumor–associated macrophages have been reported to be the major immune cell component of the TME, engaging interaction with cancer cells, T cells, B cells, endothelial cells and CAFs [39]. Elevated TAM infiltration, with an M2 phenotype, has been correlated with poor prognosis in human PDAC [40]. Our IMC analysis indicates the presence of CD206^+^ macrophages infiltrating the tumor tissue, confirming a predominance in the M2 polarization of PDAC−infiltrating TAMs, but only in the engineered mouse model and not in the orthotopic one. Recently, a peculiar population of IL−1β−expressing TAMs has been identified and correlated with inflammatory reprogramming and the acquisition of pathogenic features by tumor cells [41]. While in the orthotopic model B cells are located far from tumor cells, close to endothelial, stromal and lymphatic vessels, in the KPC genetic model, B cells are instead located in proximity to the other immune cells and LVs. Interestingly, in human PDAC, B cells scatteringly infiltrate the tumor or are organized in tertiary lymphoid structures and have been shown to correlate with longer patient survival [42]. Overall, this analysis reveals the complexity of the pancreatic TME and confirms some differences between the two pre–clinical models. The orthotopic one displayed more infiltrating immune cells and a capsule of stromal cells around the growing mass, reflecting the acute inflammatory response. By contrast, the spontaneous PDAC model displayed less abundant immune cells interspersed among stromal cells, which are also more often in proximity to epithelial and tumor cells, highly resembling a chronic inflammatory reaction typical of cancer disease. Indeed, a marker associated with constant matrix remodeling, such as uPAR, is also highly expressed not only on stromal cells but also on infiltrating mono/macrophages that reach the inner mass.

From the technical point of view, the majority of the IMC panel reported in the literature has been validated for the FFPE tissue section. The use of frozen tissue sections has some advantages and drawbacks. Compared to FFPE, frozen sections are generally thicker, limiting the accuracy of cell segmentation due to cell–cell overlap. In addition, reduced tissue dehydration before sample freezing can worsen the tissue morphology. To overcome these limitations, specific attention needs to be paid to the cell segmentation procedure: different algorithms are available and the choice of the proper segmentation pipeline can be based on the type of tissue of interest [43]. On the other side, the staining procedure is more efficient compared to the FFPE sample since it increases the number of antibodies available and their possible combination within the same IMC panel. 

IMC has been largely applied in the field of molecular oncology [21]. Most of these studies are mainly focused on reporting the possibility of applying IMC analysis to different cancer subtypes to describe the composition of the tumor microenvironment, paving the way for future additional works aimed at investigating more biological or clinical questions. These studies are particularly interesting since they facilitate the development of IMC applications in the oncology field by providing panels of antibodies usable for IMC analysis [44,45,46,47]. However, this technique has been mostly applied to human samples and only a few studies reported the use of IMC technology on mouse models of carcinogenesis [23,26]. To our knowledge, this is the first study reporting the validation of an IMC antibody panel for the investigation of a mouse model of pancreatic cancer. The 28 markers we have selected allow the identification of distinct immune, stromal and epithelial cell subpopulations, providing an overview of the tumor microenvironment and laying the foundation for further IMC analyses of murine pancreatic cancer models. Importantly, this panel can be expanded with additional markers, based on specific biological and/or clinical questions. During the setting up of the panel, the choice of targets is crucial, to have enough markers for a proper cell segmentation and a comprehensive clustering of the different cell populations in tissue. Our data showed that the IMC panel we validated is effective for proper cell segmentation and phenotypic clustering, even in tissue characterized by a high cell density, such as the orthotopic model of PDAC. In addition, the combination of phenotypic clustering, neighborhood analysis and the tissue localization of cell markers and clusters allows a better investigation of the complexity of the TME.

In conclusion, we validated a 28–antibody panel to investigate the TME of pancreatic cancer in mouse models. Our panel is designed to identify the main stromal components and immune cell populations together with the identification of tumor cells for downstream single–cell analysis. Moreover, additional metal isotope–conjugated antibodies can be added to customize the panel based on specific research needs, to increase the knowledge of PDAC complexity and to identify new prognostic and therapeutic approaches for the treatment of the disease. IMC could also complement current efforts aimed at defining human pancreatic cancer molecular profiles, which have collectively led to the identification of the classical and squamous subgroups [48]. So far, transcriptomic data have been primarily used. The combination with multi–dimensional analyses of the tumor immune microenvironment by IMC could be exploited to validate molecular signatures as clinically relevant biomarkers.

## 4. Materials and Methods

### 4.1. Animal Maintenance and Tissue Samples

All mice were on a C57B/6 genetic background, maintained in a specific–pathogen–free facility in individually ventilated cages, and given ad libitum access to food and water. Procedures involving animals conformed to institutional guidelines, in compliance with national (D.L. N.116, G.U., suppl. 40, 18-2-1992 and N. 26, G.U. 4 March 2014) and international laws and policies (EEC Council Directive 2010/63/EU, OJ L 276/33, 22 September 2010; National Institutes of Health Guide for the Care and Use of Laboratory Animals, US National Research Council, 2011). Animal procedures were approved by the Italian Ministry of Health (authorization 158/2011 14 September 2011, ID 6B2B3, approval No. 13/2021-PR, authorization 428/2023-PR). 

### 4.2. Panc02 Orthotopic Model of Pancreatic Cancer and Tissue Preparation

The Panc02 cell line was cultured in RPMI–1640 medium (Euroclone, Pero, Italy), supplemented with 10% fetal bovine serum (FBS, Euroclone, Pero, Italy), 1% Glutamine (Lonza, Basel, Switzerland), 1% Pen/Strept (Euroclone, Pero, Italy). On the day of the injection, cells were detached with Trypsin/EDTA (Lonza, Basel, Switzerland), washed, and resuspended in saline solution. Eight– to twelve–week–old mice were anesthetized by intraperitoneal injection of ketamine (100 mg/mL) and xylazine (10 mg/kg). Via abdominal midline incision, the stomach was gently exteriorized to expose the head of the pancreas. Subsequently, 1 × 10^6^ Panc02 cells, in a volume of 30 μL, were injected into the head of the pancreas. The peritoneum and abdominal wall were closed with individual surgical sutures. At day 21 after tumor cell injection, mice were sacrificed by CO_2_ inhalation. Tumor samples were fixed overnight in 2% PFA and then included in optimal cutting temperature (OCT). Subsequently, 10 µm–thick sections were cut with a cryostat (Leica, Wetzlar, Germany).

### 4.3. KPC Mouse Model of Pancreatic Cancer and Tissue Preparation

KPC mice models were generated by crossing double–mutated *LSL*–*Kras^G12D^*; *LSL*–*Trp53^R172H^* with C57BL/6 mice expressing Cre recombinase under the *Pdx*–*1* promoter. Mice were screened using tail DNA amplified by specific primers to the Lox–P cassette flanking Kras, Trp53 mutated and wild–type, and Cre recombinase genes as previously described [35]. Pancreases were resected from KPC mice at 20 weeks of age, embedded in tissue–Tek O.C.T. compound, and immediately frozen in liquid nitrogen. Subsequently, they were sliced into 10 µm sections with Cryostar NX50 (Thermo Fisher Scientific, Waltham, MA, USA).

### 4.4. Antibodies and Metal Conjugation

The complete list of metal–tagged antibodies is reported in Table 1. Anti–Ly6G, PanCK E–cadherin, Collagen I, CD44, and CD11b metal–tagged antibodies were purchased from Standard Biotools. All the remaining antibodies were conjugated using the Maxpar^®^ X8 Antibody Labeling Kit (Standard Biotools, San Francisco, CA, USA), according to the manufacturer’s instructions, and resuspended in PBS^2+^ (pH = 7.4) and 0.05% NaN_3_.

### 4.5. Tissue Labelling for Imaging Mass Cytometry

Frozen sections were washed in PBS^2+^ for 10 min. Tissue sections from KPC mice were fixed in 4%PFA, for 5 min at room temperature in a dark chamber (Panc02–derived tumors were already fixed before the OCT embedding, as previously reported). Subsequently, sections were incubated with the antibody mix, diluted in PBS^2+^, 2% BSA, 5% normal mouse (Biosera, Cholet, France)/rat(Sigma–Aldrich, St. Louis, MO, USA)/rabbit(Dako, Santa Clara, CA, USA)/goat(Sigma–Aldrich, St. Louis, MO, USA) serum, 0.3% Triton–X (Sigma), o/n at 4 °C. After the incubation, sections were washed 4 times, 5 min each, in PBS^2+^ 0.05% Tween–20 (Merck, Burlington, MASS, USA). For nuclear staining, tissues were then incubated with 0.6 µM Ir191/193 (Standard Biotools, San Francisco, CA, USA) in PBS for 30 min at RT. After incubation, tissue sections were washed 3 times, 3 min each, in PBS^2+^ 0.05% Tween20. Finally, sections were washed in ultrapure H_2_O to remove salt leftovers and airdried.

### 4.6. Data Acquisition with Imaging Mass Cytometry

Images were acquired with a Hyperion Imaging System (Standard Biotools, San Francisco, CA, USA), according to the manufacturer’s instructions. For the orthotopic mouse model, 4 mice were used and the following ROIs per mouse were acquired: mouse 1: core 2 ROIs, margin 1 ROI; mouse 2: core 2 ROIs, margin 2 ROIs; mouse 3: core 1 ROI, margin 3 ROIs; mouse 4: core 3 ROIs, margin 3 ROIs. For the genetic models, 2 mice were used and the following ROIs per mouse were selected: KPC1: 4 ROIs; KPC2: 5 ROIs

Selected ROIs were laser ablated with a UV laser, with a frequency of 200 Hz, at a resolution of approximately 1 μm^2^. To ensure system stability, the Hyperion Imaging System was routinely calibrated following the manufacturer’s instructions.

### 4.7. Data Analysis

IMC image analysis was performed using a custom pipeline based on the original pipeline reported in https://github.com/BodenmillerGroup/ImcSegmentationPipeline [19], accessed on 27 December 2023.

Single–channel images were extracted from mcd files using MCDviewer (version 1.0.560.6) software (Standard Biotools, San Francisco, CA, USA). Hot pixel removal was performed with the ImageJ Remove Ouliers plugin (radius = 2, threshold = 50) [49] and saved in a metadata file. Tiffs substacks containing the complete list of channels relevant for segmentation and cell classification were created using the R custom pipeline and normalized at the top 99% percentile of expression (or at least at an intensity value of 10 dual counts). For each channel, low–intensity thresholds were manually settled based on visual inspection. Single–pixel classification was performed with Ilastik (v1.3.3post3) [50] to generate probability maps for nuclei, cytoplasm, and background. Then, segmentation masks were created using CellProfiler v4.2.1 [51]. Mean channel intensity inside each cell and shape parameters were calculated with the computeFeatures function from the R EBImage package (version 4.36) [52]. The following criteria were used to exclude objects in the mask that were likely to represent debris or segmentation errors: area larger than 500 µm^2^, mean intensity higher than 2 in all the markers, and lower than 0.01 in those markers used for UMAP analysis. No more than 2% of the cells were discarded based on these criteria. Inverse hyperbolic sine was used to rescale the data before normalization between 1% and 99% of the overall signal. An R implementation of the UMAP algorithm (https://CRAN.R-project.org/package=umap, accessed on 27 December 2023, version 0.2.8) and *phenograph* (version 0.99) [53] (k = 100) were used to cluster the data and classify cellular subpopulations. For each model, UMAP was performed over all the segmented cells obtained from all the acquired ROIs. Clusters were assigned to different cellular populations based on specific markers, as described in Section 2. Data were visualized with *dittoSeq* (version 1.6.0) and *ggplot2* (version 3.4) packages [54]. For heatmap representation, data were averaged per cluster and normalized per row. 

Cell–to–cell proximity score was determined using the *testInteractions* function from the imcRtools package (version 1.9.0) [19]. Briefly, cells were considered as neighbors if their distance, calculated using the 3D interaction Fiji plugin (version mcib3D 4.1.5) [55] between their borders, was less than 30 μm. For each ROI, a permutation test (*n* = 1000) of the cell labels was performed to assign a *p*–value to each couple of neighboring cell clusters. Interactions between clusters with *p*–value < 0.01 were assigned a score +/–1 according to their enrichment or depletion compared to randomized interactions, while a score of 0 was assigned to non–significant interactions. The resulting scores, averaged over all the ROIs, were represented in heatmaps.

### 4.8. Image Processing and Statistical Analysis

Representative images were prepared using Fiji (ImageJ, version 1.54f) software. Graphs and statistical analysis were performed with GraphPad Prism software (version 9.0.2). Two–way ANOVA plus Šídák’s multiple comparisons test was applied. A *p*–value < 0.05 was considered statistically significant. 

## Figures and Tables

**Table 1:** Imaging mass cytometry antibody panel.

Target	Clone	Metal	Source
Ly6G	1A8	141Pr	Standard Biotools (San Francisco, CA, USA)
Vimentin	D21H3	143Nd	Cell Signaling (Danvers, MA, USA)
MHC–II	M5/114.15.2	145Nd	Invitrogen (Waltham, MA, USA)
CD45	30-F11	147Sm	Invitrogen
PanCK	AE1/AE3	148Nd	Standard Biotools
Fibrinogen	Polyclonal	149Sm	Dako
αSMA	1A4	151Eu	Sigma
CK19	EPNCIR127B	152Sm	Abcam (Cambridge, UK)
F4/80	Cl:A3-1	153Eu	BioRad (Hercules, CA, USA)
PDGFRα	Polyclonal	154Sm	R&D (Sunnyvale, CA, USA)
uPAR	Polyclonal	155Gd	R&D
PDGFRβ	Polyclonal	156Gd	R&D
E–cadherin	24E10	158Gd	Standard Biotools
CD3	17A2	159Tb	Invitrogen
PV1	MECA-32	160Gd	BD Pharmigen (San Diego, CA, USA)
CD4	GK1.5	161Dy	Invitrogen
CD8	53-6.7	163Dy	Invitrogen
Lyve–1	Polyclonal	164Dy	Abcam
Desmin	Y66	165Ho	Abcam
CD31	2H8	166Er	Merck Millipore (Burlington, MA, USA)
Collagen IV	Polyclonal	167Er	BioRad
CD206	Polyclonal	168Er	Abcam
Collagen I	Polyclonal	169Tm	Standard Biotools
CD44	IM7	171Yb	Standard Biotools
CD11b	M1/70	172Yb	Standard Biotools
ZO−1	1A12	173Yb	Invitrogen
CD103	Polyclonal	175Lu	R&D
B220	RA3-6B2	176Yb	Invitrogen

## Data Availability

The data presented in this study are available on request from the corresponding authors.

## Supplementary Materials

The following supporting information can be downloaded at: https://www.mdpi.com/article/10.3390/ijms25031389/s1.
