# Supplementary material for: Multiplexed Imaging Mass Cytometry Analysis in Preclinical Models of Pancreatic Cancer
Source: Int J Mol Sci. 2024 Jan 23;25(3):1389. doi: 10.3390/ijms25031389 (PMC10855072; doi:10.3390/ijms25031389)
Supplement: Supplementary file 1 [file ijms-25-01389-s001.zip › ijms-2823052-supplementary.pdf]

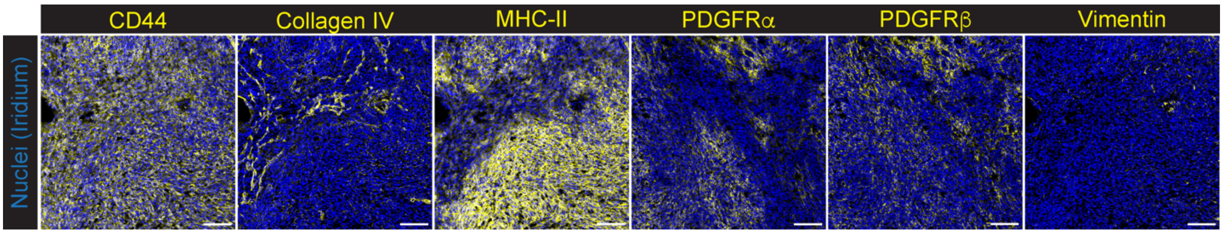

Supplementary Figure S1: Expression of different markers at the tumor–stroma interface of the orthotopic PDAC model by IMC. Nuclei (Ir, blue) and marker expression (yellow) are shown. Bar, 100μm

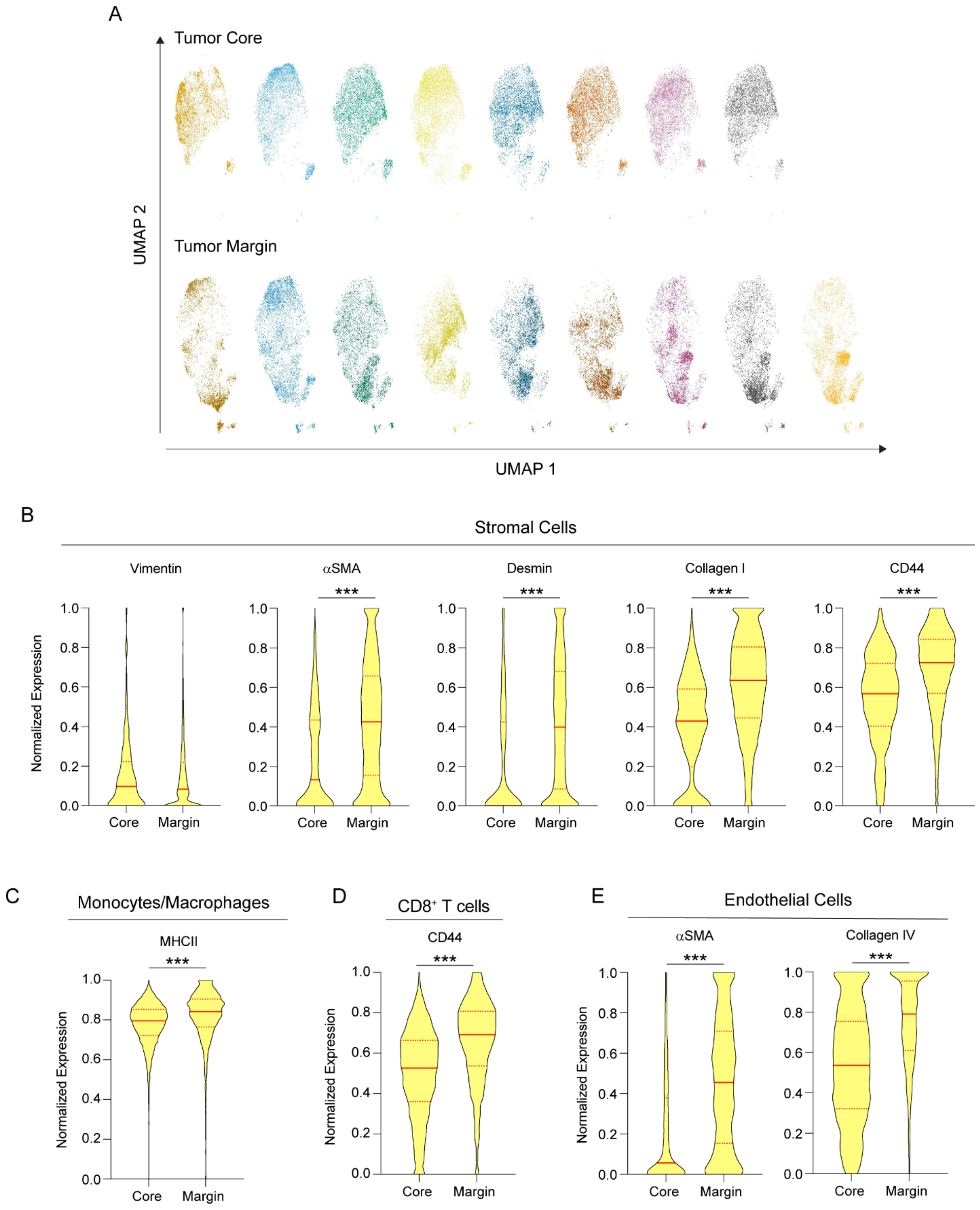

Supplementary Figure S2: Single–cell distribution in the orthotopic PDAC model. A) Graphs show the distribution of segmented cells from single acquired ROIs in the UMAP reduced space. UMAP was performed over all the cells from orthotopic model. *n*= 3 to 6 ROIs for each mouse (*n*=4); Total acquired

ROIs: core: n=8; margin: n=9. **B)** Violin plots showing the difference in the expression of Vimentin, αSMA, Desmin, Collagen I and CD44 in the stromal cells between tumor core and margin. n= 3 to 6 ROIs for each mouse (n=4); Total acquired ROIs: core: n=8; margin: n=9; Mann–Whitney test: *** $p<0.001$ **C)** Violin plots showing the difference in the expression of MHC–II in monocytes/macrophages between tumor core and margin. n= 3 to 6 ROIs for each mouse (n=4); Total acquired ROIs: core: n=8; margin: n=9; Mann–Whitney test: *** $p<0.001$ **D)** Violin plots showing the difference in the expression of CD44 in $CD8^+$ T cells between tumor core and margin. n= 3 to 6 ROIs for each mouse (n=4); Total acquired ROIs: core: n=8; margin: n=9; Mann–Whitney test: *** $p<0.001$ **E)** Violin plot showing the difference in the expression of αSMA and Collagen IV in endothelial cells between tumor core and margin. n= 3 to 6 ROIs for each mouse (n=4); Total acquired ROIs: core: n=8; margin: n=9; Mann–Whitney test: *** $p<0.001$

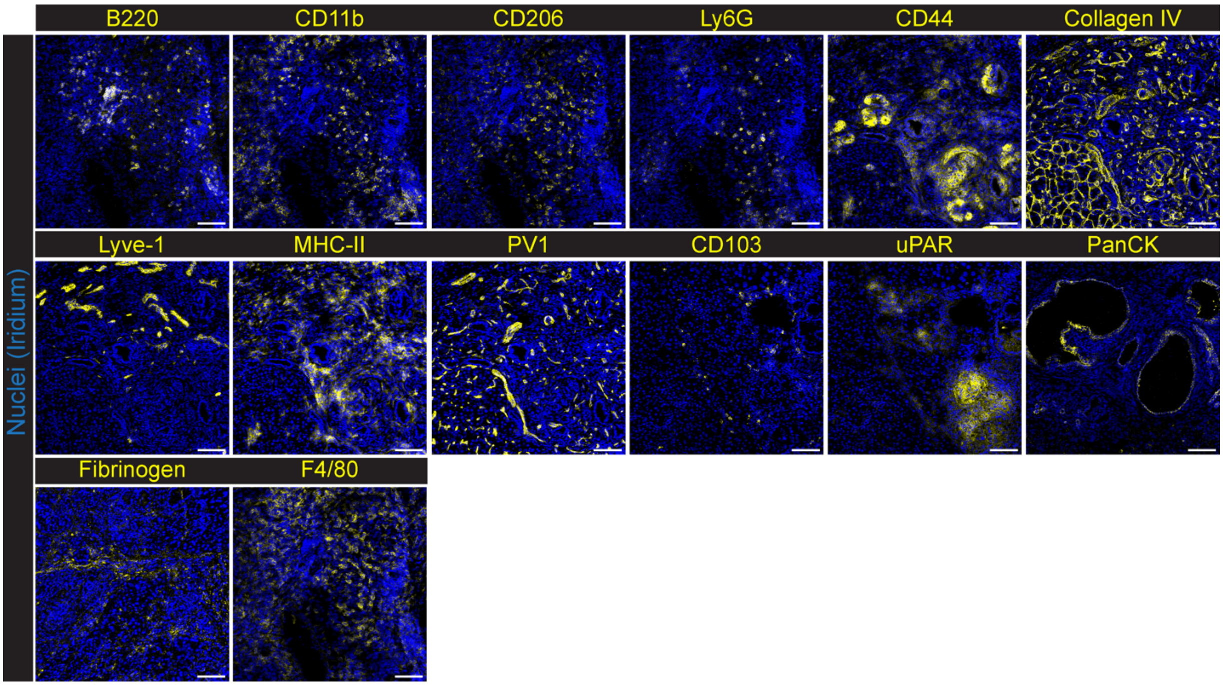

Supplementary Figure S3: Expression of different markers in the genetic KPC model by IMC. Nuclei (Ir, blue) and marker expression (yellow) are shown. Bar, 100µm

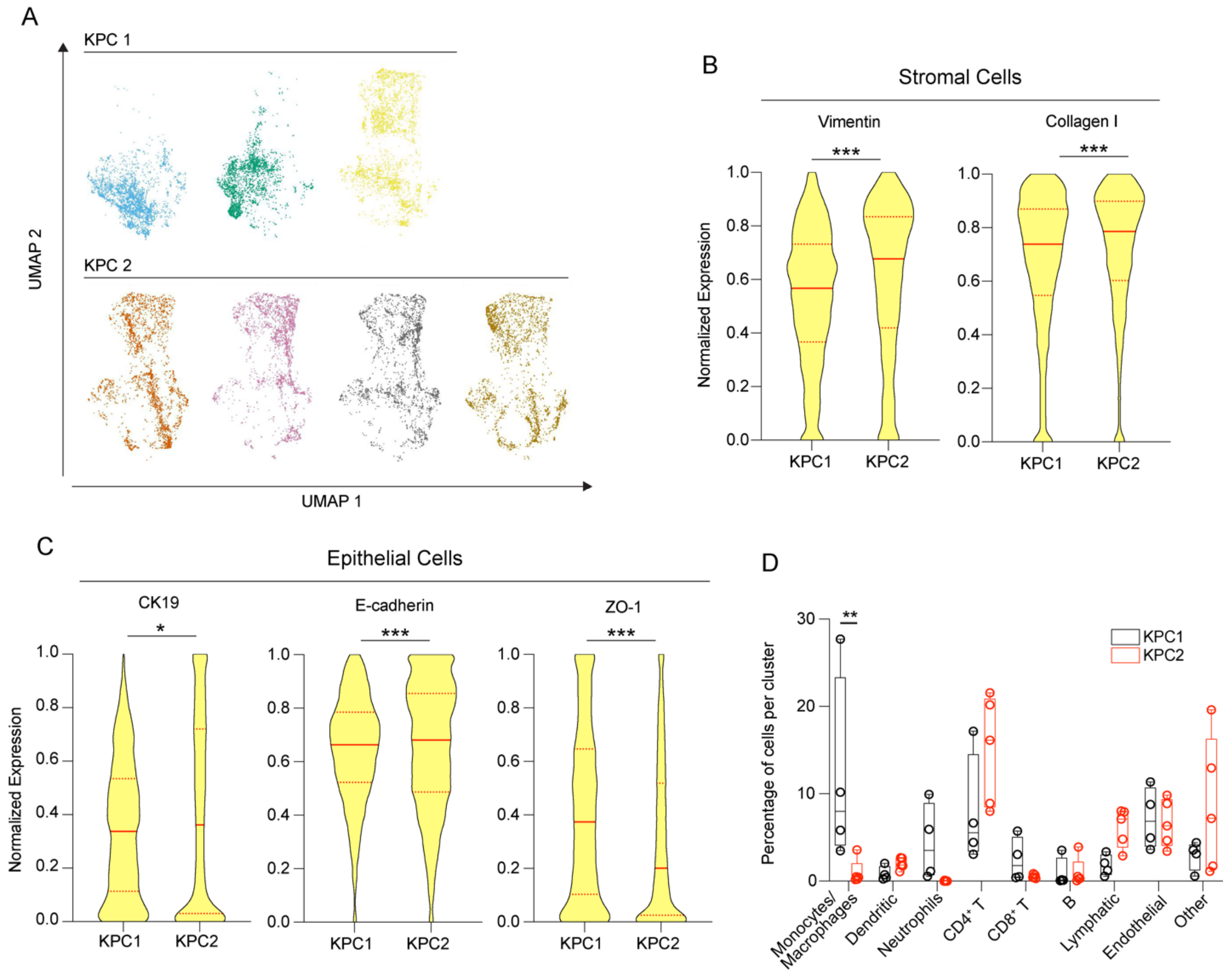

Supplementary Figure S4: Single cell distribution in the genetic KPC model. A) Cell distribution in the UMAP reduced space for each single ROI in the KPC model. **B)** Violin plot showing the difference in the expression of Vimentin and Collagen I in stromal cells between KPC1 and KPC2 tumor. *n*= 4 to 5 ROIs for each mouse (*n*=2). Total acquired ROIs, KPC1, *n*=4; KPC2, *n*=5. Mann–Whitney test: *** *p<0.001.* **C)** Violin plot showing the difference in the expression of CK19, E–cadherin and ZO–1 in epithelial cells between KPC1 and KPC2 tumor. *n*= 4 to 5 ROIs for each mouse (*n*=2). Total acquired ROIs, KPC1, *n*=4; KPC2, *n*=5. Mann–Whitney test: * *p<0.05*, *** *p<0.001.* **D)** Box plot shows the difference in the number of cells per cluster between KPC1 and KPC2 mice. Dots represent single ROI abundances. *n*= 4 to 5 ROIs for each mouse (*n*=2). Total acquired ROIs, KPC1, *n*=4; KPC2, *n*=5. Two–way ANOVA plus Šídák's multiple comparisons test; ** *p<0.01.* **C)** Violin plots showing the difference in the expression of CD44 in $CD8^+$ T cells. Every dot corresponds to a single cell. *n*= 4 to 5 ROIs for each mouse (*n*=2). Total acquired ROIs, KPC1, *n*=4; KPC2, *n*=5. Mann–Whitney test: *** *p<0.001*
